# The Evolution of Character

**Published:** 1915-07

**Authors:** 

**Affiliations:** San Antonio


### The Evolution of Character.

It is a long step from Hippocrates and Galan to our present-day interpretation of psychological phenomena. Yet, the nature of human development seems to require this slow evolutionary process. Character and temperament occupied the thoughts and discussions of Aristotle and his contemporaries as wholly as it does our teachers of the present day. The ancients, however, had to rely on empirical knowledge, whereas our present-day scholars have the record of social culture, for two thousand years, from which to draw.

The antecedents of character, or the successive steps by which our present knowledge has been evolved, is particularly interesting. Hippocrates taught that the elements of matter consisted in the form of air, fire, water and earth; that temperaments were either sanguine, phlegmatic, melancholic or chloric, and corresponded to the seasons of the year. He devised the doctrine of the humors—blood, bile, phlegm and black bile. All disease he attributed to some affection of one of these. Likewise, he ascribed the different temperaments to these humors. Thus, he thought that cheerfulness resided in the blood, laziness in the phlegm, testiness in the bile and low spirits (or depression) in the black bile.

Astrology was the next step in the attempt to determine character, and it was further utilized to foretell coming events. The time of one's birth, as related to the influence of the planets, determine the character of his temperament, and preordained his destiny. Thus, to be born under Jupiter's wing assured a jovial disposition, while Saturn foretold a gloomy existence.

As culture progressed, some minds in advance of the masses, with greater enthusiasm, perhaps, than scientific knowledge, advanced pseudo-scientific ideas that wielded, at the time, tremendous influence. The aim always was the control of Fate through some insight or revelation. Such was the doctrine of physiognomy, as developed by Lavater, in the latter part of the eighteenth century. He held that mental traits were conditioned by bodily composition and could be read, therefore, by the contour of the face and form. Following Lavater and his adherents was the more plausible claims of Gall, pronouncing his explanation of judging character by certain physical forms of the head, or "bumps," popularly known as phrenology. More recently, and what still remains in favor, is palmistry, having in view the same aim of unlocking the door of the future.

All of these theories were accepted in their time; each had some claims to plausibility, in the absence of more exact knowledge, but being fallacious, like other theories without scientific facts, they have all died out, except in the minds of those who are still strongly inclined towards occultism or mysticism.

Psychology, while empirically suggested by the pathfinders of knowledge, at present takes its place side by side with other sciences. In its interpretation of brain power, we learn the true nature of character and temperament. We now know that the brain develops as a whole, and not in parts as was supposed by the phrenologists, and that it is capable of any degree of mental development, consistent with its inherent endowment. We also know that this development is determined largely by the environment and training to which the brain is subjected, and it is not manifested in any peculiar lines of the face or form. Such peculiarities of features, as are ascribed to mental character, are the result of general inheritance. The test of character is the volition of the will. Any forecast of one's actions can only be suggested by a test of his mentality, considered in connection with his environment. The faculty one person has of reading the character of another depends upon the degree of his sensitiveness to respond to psychological forces operating in his sphere of influence.

While modern psychology recognizes physiology as its basis, it has remained for a Yale professor, Dr. Frost, to discover the mechanism by which this relationship operates. Dr. Frost has shown how the sensorimotor arc actually controls habit formation. This is done through the mechanism which he calls the synapse. This organ is the junction of the ingoing and outgoing nerve fibers. To get from a sensory nerve to a motor nerve, the impulse must pass through this point, and when once passed it cannot be returned, though it may pass any number of them. It further determines in which direction the impulse will be sent. In this action it resembles a trap door, or a switchboard at a telephone.

It is a law that nervous impulse follows the path of least resistance. To illustrate: In the case of a drunkard, the synapse (a) offers the least resistance, hence the man drinks. If he is a total abstainer, the synapse (a) offers a greater resistance than (b), hence he refrains from drinking. In other words, the formation of a habit is the breaking down of the natural resistance afforded at one synapse, and the raising of the resistance afforded at another. To reform a habit requires a readjustment of the synapic resistance, and the process is the reverse of this. The synapse of the greatest resistance is broken down, and the one that was previously weak is strengthened.

The nerve trunk transmitting the impulse is merely a conducting wire and is unfatiguable. But the synapse easily becomes fatigued by repeated stimulation. When it wears out, there results an increased resistance offered to the impulse so that either the stimulant must be increased to maintain the same action, or the muscular activity will be weakened. The synapses will not open to weak impulses, and they exhibit a lively effect from the use of poisons and narcotics.

So much for the mechanics of habit formation. There must be the psychic element to operate conjointly with this mechanism. For example, to reform an old habit requires a recognition, first, of the desirability and necessity for making the change. And then, when the old channel is closed, there must be provided a legitimate outlet for the energy thus encased; i. e., a new habit must be instituted to replace the old. Secondly, it is important that the new energy be exerted at the proper time. The psychological moment must be observed. It is much easier to accomplish the change when all predisposing conditions are favorable. When the system is tired and nervous it is more difficult to establish resistance, as illustrated when temptation is offered at a time when the body is fatigued or the feelings depressed.

Thus it appears that by modifying the synapse you change consciousness. By varying the synapic resistance you modify or reverse behavior. All nervous action, in other words, consciousness, in fact, is controlled or modified through this synapic action.

It is comparatively easy to form a new habit. It simply requires that the first experience be agreeable and the path of stimulation is quickly established. But, as above pointed out, to change a habit requires the exercise of will power, persistently applied in order to change the synapic resistance.

